# Cost Effectiveness of DCISionRT for Guiding Treatment of Ductal Carcinoma in Situ

**DOI:** 10.1093/jncics/pkaa004

**Published:** 2020-01-31

**Authors:** Ann C Raldow, David Sher, Aileen B Chen, Rinaa S Punglia

**Affiliations:** p1 Department of Radiation Oncology, David Geffen School of Medicine at UCLA, Los Angeles, CA, USA; p2 Department of Radiation Oncology, University of Texas Southwestern, Dallas, TX, USA; p3 Department of Radiation Oncology, MD Anderson Cancer Center, Houston, TX, USA; p4 Department of Radiation Oncology, Dana-Farber Cancer Institute, Boston, MA, USA

## Abstract

The DCISionRT test estimates the risk of an ipsilateral breast event (IBE) in patients with ductal carcinoma in situ (DCIS) as well as the benefit of adjuvant radiation therapy (RT). We determined the cost-effectiveness of DCISionRT using a Markov model simulating 10-year outcomes for 60-year-old women with DCIS based on nonrandomized data. Three strategies were compared: no testing, no RT (strategy 1); test all, RT for elevated risk only (strategy 2); and no testing, RT for all (strategy 3). We used utilities and costs from the literature and Medicare claims to determine incremental cost-effectiveness ratios and examined the number of women irradiated per IBE prevented. In the base-case scenario, strategy 1 was the cost-effective strategy. Strategy 2 was cost-effective compared with strategy 3 when the cost of DCISionRT was less than $4588. The number irradiated per IBE prevented were 8.37 and 15.46 for strategies 2 and 3, respectively, relative to strategy 1.

There has been growing interest in the use of genomic assays to improve treatment selection for patients with ductal carcinoma in situ (DCIS). However, genomic tests are costly, and the best strategy for their use is uncertain. We previously reported on the cost-effectiveness of using the Oncotype DX DCIS Score, a test that uses tumor biology to predict recurrence risk (1). DCISionRT is another test that is predictive of an individual patient’s benefit from radiation therapy (RT) (2). DCISionRT provides 10-year total and invasive recurrence risks after breast-conserving surgery with or without adjuvant RT. We analyzed the cost-effectiveness of the DCISionRT test to guide treatment of DCIS.

Bremer and colleagues published a development and cross-validation study of DCISionRT, a prognostic and predictive test for DCIS^3^. In the study population, which consisted of 526 patients, 59% of patients underwent adjuvant RT and 29% received adjuvant hormonal treatment. Detailed patient characteristics are found in Table 1 of the Bremer study. Using this nonrandomized study, we developed a Markov model to characterize patient health states after lumpectomy with either surgery alone (OBS) or surgery with RT to simulate 10-year outcomes for 60-year-old women. Model cycle length was 1 year; the 10-year recurrence risks with and without RT were derived from the Bremer study and converted to 1-year probabilities. The model was analyzed using TreeAge Pro 2018 (Williamstown, MA).

**Table 1.** Incremental cost-effectiveness ratios for the treatment strategies*

Strategy	Cost	Incremental cost	Effectiveness	Incremental effectiveness	Incremental cost/effectiveness	Cost/effectiveness	Dominance
Excluding dominated (Strategy 2)							
Strategy 1	1695.865	—	8.570992	—	—	197.861	Undominated
Strategy 3	12 118.17	10 422.31	8.590681	0.019688	529 365.8	1410.618	Undominated
All							
Strategy 1	1695.865	—	8.570992	—	—	197.861	Undominated
Strategy 3	12 118.17	10 422.31	8.590681	0.019688	529 365.8	1410.618	Undominated
Strategy 2	12 224.17	106.001	8.583273	−0.00741	−14 308.9	1424.186	Absolutely dominated
All referencing common baseline							
Strategy 1	1695.865	—	8.570992	—	—	197.861	Undominated
Strategy 3	12 118.17	10 422.31	8.590681	0.019688	529 365.8	1410.618	Undominated
Strategy 2	12 224.17	10 528.31	8.583273	0.01228	857 336.1	1424.186	Absolutely dominated
All by increasing effectiveness							
Strategy 1	1695.865	—	8.570992	—	—	197.861	Undominated
Strategy 2	12 224.17	—	8.583273	—	—	1424.186	Absolutely dominated
Strategy 3	12 118.17	—	8.590681	—	—	1410.618	Undominated

* When one strategy is both less effective and more expensive, that strategy is absolutely dominated by the other strategies. Extended dominance occurs when the incremental cost-effectiveness ratio for a given treatment alternative is higher than that of the next, more effective, alternative. RT = radiation therapy; Strategy 1 = no testing, no RT; Strategy 2 = test all, RT only for elevated risk; Strategy 3 = no testing, RT for all.

Three treatment strategies were compared. In strategy 1, no patients were tested with the DCISionRT Score, and no one underwent initial RT. In strategy 2, all the patients were tested, and those with elevated-risk scores underwent RT. In strategy 3, no patients were tested, and all patients underwent adjuvant RT.

Supplementary Table 1 (available online) depicts the probabilities, costs, and utilities used in the study (3,4). We employed methods similar to those previously published (1). Hypothetical patients began in a no evidence of disease (NED) state, having undergone lumpectomy with or without adjuvant RT. Patients then remained in the NED states or proceeded to an ipsilateral breast event (IBE) state. Life tables were used to calculate the probability of entering the death state (5). The proportion of women with low- and elevated-risk groups and their expected IBE risks were based on Bremer et al. (3). A payer (Medicare) perspective was used to derive 2019 costs (1). Utilities and costs were discounted 3% annually.

We report the incremental cost-effectiveness ratio when one strategy is more effective but more costly (or less effective but less costly) compared with strategy 1. We used a willingness-to-pay threshold of $100 000/quality-adjusted life-year. We also calculated the number of women needed to irradiate (NNI) per IBE and invasive breast cancer prevented for each strategy compared with the reference strategy 1.

Strategy 1, 2, and 3 were associated with quality-adjusted life-years of 8.57, 8.58, and 8.59, and mean per-person costs of $1696, $12 224, and $12 118 respectively (Table 1). None of the treatment strategies, including the one incorporating the DCISionRT Score, were cost-effective compared with the reference strategy 1.

The percentages of patients with IBE at 10 years were 14.6%, 9.1%, and 8.6% for strategies 1, 2, and 3, respectively. The percentages of patients with an invasive recurrence at 10 years were 9.1%, 6.3%, and 5.8% for strategies 1, 2, and 3, respectively.

The utilities of the NED-RT and NED-OBS health states were assumed to be the same (0.90) in the base case (4). To model anxiety associated with not receiving RT, we performed a one-way sensitivity analysis, varying the utility of the NED-OBS state (Supplementary Figure 1A available online). Strategy 3 was cost-effective when the utility of the NED-OBS state was less than 0.890. Compared with strategy 3, strategy 2 was the cost-effective strategy when the utility of the NED-OBS state was greater than 0.8996.

To model anxiety and side effects associated with receiving RT, we performed a one-way sensitivity analysis varying the utility of the NED-RT state (Supplementary Figure 1B available online). Strategy 3 became cost-effective when the utility of the NED-RT state was greater than 0.909. Compared with strategy 3, strategy 2 was the cost-effective strategy when the utility of the NED-RT state was less than 0.897.

We performed a one-way sensitivity analysis to determine the effect of varying the cost of RT. Strategy 3 was the favored strategy compared with both strategies 1 and 2 when the cost of RT was less than $2410. Similarly, we performed a one-way sensitivity analysis to determine the effect of varying the cost of DCISionRT (Figure 1). Although strategy 1 remained the cost-effective strategy regardless of DCISionRT cost, strategy 2 was the favored strategy compared with strategy 3 when the cost of the test was less than $4588.

**Figure 1.**
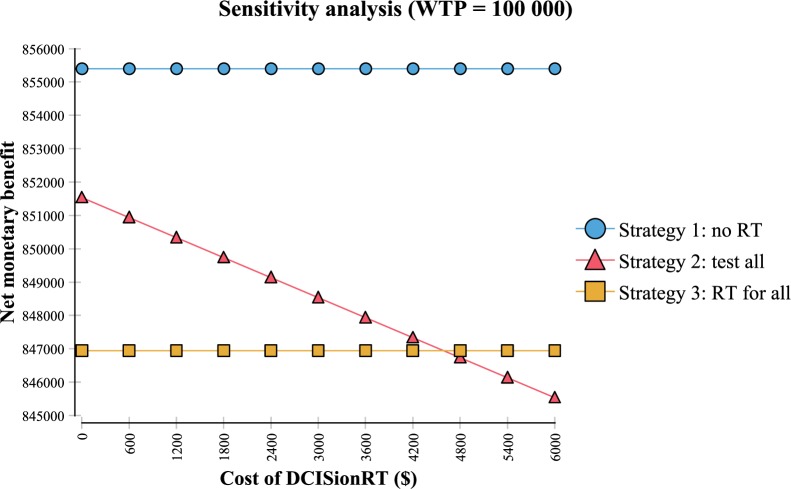
Sensitivity analysis varying the cost of DCISionRT using a willingness-to-pay (WTP) threshold of $100 000/QALY. The net monetary benefit (NMB) of an intervention is the difference between the monetary value of total expected QALYs (WTP multiplied by expected QALYs) and total expected costs [NMB = (WTP × ΔQALYs) − Δcosts]. If the NMB of one intervention exceeds the NMB of a second intervention, the first intervention is cost-effective compared with the second intervention. RT = radiation therapy.

The NNI per IBE prevented was 8.37 and 15.46 for strategies 2 and 3, respectively. The respective estimates for the NNI per invasive breast cancer prevented were 16.82 and 28.64. Compared with strategy 3, strategy 2 minimized the number of women undergoing RT per IBE prevented.

In summary, we found that strategy 1 was the cost-effective strategy for DCIS patients. However, decreasing the utility of the NED-OBS state by very slightly (<0.01) below the utility the NED-RT state made strategy 3 cost-effective. These results confirm the importance of engaging patients in decision-making and gaining an understanding of their preferences. The benefit of RT depends on the trade-off between the anxiety and IBE risk vs the time commitment, anxiety, and side effects of RT.

However, when comparing strategy 2 with strategy 3, strategy 2 was the cost-effective strategy when the cost of the test was less than $4588. Compared with giving RT to all women with DCIS, strategy 2 with DCISionRT minimized the number of women undergoing RT per IBE prevented.

We acknowledge that our Markov model is based on nonrandomized data from one study of two cohorts; however, sensitivity analyses were performed to account for uncertainty of key variables, including utilities of NED states and DCISionRT risk estimates. Utilities were derived from a study explicitly evaluating patient utility values after treatment of DCIS. Although completed in 2005, standard treatment has not substantially changed since the publication of this study. The publication of further validation cohorts that were presented at the San Antonio Breast Cancer Symposium in 2016 and 2017 should allow an updated analysis based on a much larger cohort of patients from more diverse settings. This would allow a much more rigorous assessment of the cost effectiveness of the DCISionRT assay.

## Notes

Ann C. Raldow has received consulting fees from Intelligent Automation, Inc. All other authors have no conflicts of interest to disclose.

## Supplementary Material

pkaa004_Supplementary_Data
