# Supplementary material for: Cost Effectiveness of DCISionRT for Guiding Treatment of Ductal Carcinoma in Situ
Source: JNCI Cancer Spectr. 2020 Jan 31;4(2):pkaa004. doi: 10.1093/jncics/pkaa004 (PMC7083239; doi:10.1093/jncics/pkaa004)

Supplementary Table 1. Markov Model Costs, Utilities and Probabilities

Health State	Utility[4]
Lumpectomy and RT without recurrence	0.90
Lumpectomy and RT with invasive local recurrence salvaged with mastectomy	0.81
Lumpectomy and RT with DCIS recurrence salvaged with mastectomy	0.88
Lumpectomy without recurrence	0.90
Lumpectomy with invasive local recurrence salvaged with mastectomy	0.84
Lumpectomy with DCIS recurrence salvaged with conservative surgery and RT	0.90
Lumpectomy with DCIS recurrence salvaged with mastectomy	0.89
Death	0
Procedure	Cost ($)
DCISionRT test	5,435
RT after lumpectomy	11,507
Lumpectomy and RT after DCIS recurrence	15,205
Mastectomy after DCIS recurrence	6,824
Lumpectomy and RT after invasive local recurrence	16,728
Mastectomy after invasive local recurrence	7,218
States	Probability[3] (10-year)
Having an elevated-risk score	0.52
Having a low-risk score	0.48
DCIS recurrence for low risk score treated without adjuvant RT	0.04
Invasive recurrence for low risk score treated without adjuvant RT	0.04
DCIS recurrence for low risk score treated with adjuvant RT	0.04
Invasive recurrence for low risk score treated with adjuvant RT	0.03
DCIS recurrence for elevated risk score treated without adjuvant RT	0.08
Invasive recurrence for elevated risk score treated without adjuvant RT	0.15
DCIS recurrence for elevated risk score treated with adjuvant RT	0.02
Invasive recurrence for elevated risk score treated with adjuvant RT	0.09

Supplementary Figure 1. Sensitivity analyses varying the utilities of the no evidence of disease without adjuvant RT (NED-OBS) and no evidence of disease after adjuvant radiation therapy (NED-RT). The utilities of the NED-OBS and NED-RT states were the same (0.90) in the base case model. (A) Varying the utility of the NED-OBS state. (B) Varying the utility of the NED-RT state.

A.

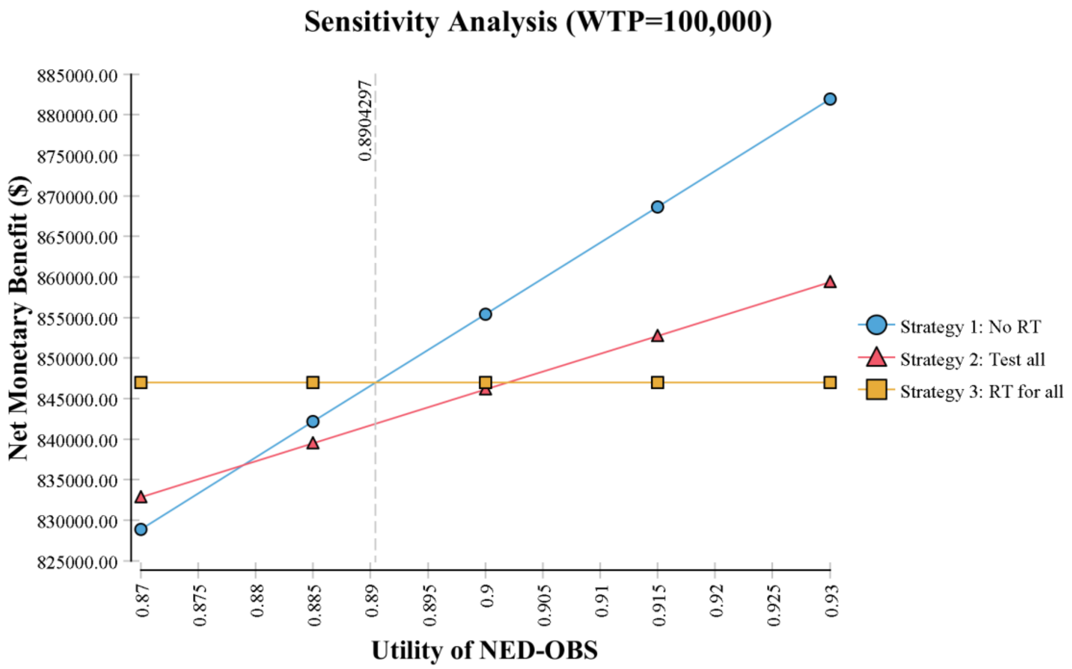

B.

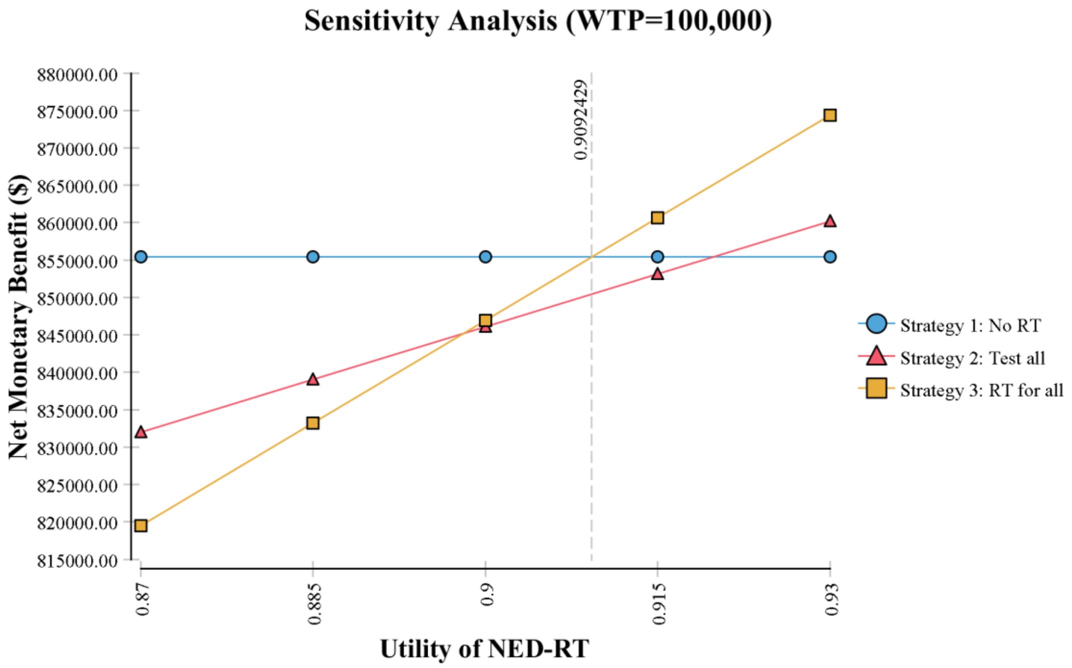

Supplement: pkaa004_Supplementary_Data [file pkaa004_supplementary_data.pdf]
